# Topical Semisolid Products—Understanding the Impact of Metamorphosis on Skin Penetration and Physicochemical Properties

**DOI:** 10.3390/pharmaceutics14112487

**Published:** 2022-11-17

**Authors:** Xuping Jin, Mohammad Imran, Yousuf Mohammed

**Affiliations:** 1 Therapeutics Research Group, Frazer Institute, Faculty of Medicine, University of Queensland, Brisbane, QLD 4102, Australia; 2 School of Pharmacy, University of Queensland, Brisbane, QLD 4102, Australia

**Keywords:** metamorphosis, in vitro permeability, rheology, microscopical analysis, critical quality attributes

## Abstract

Recently, the United States Food and Drug Administration published a series of product-specific guidance for the development of topical drugs, with in vitro options consisting of qualitative sameness (Q1) and quantitative sameness (Q2) assessment of formulations, physiochemical and structural characterization of formulations (Q3), and, potentially, in vitro drug release and permeation tests. In these tests, the topical semisolid product’s critical quality attributes (CQAs), such as rheological properties, thermodynamic activity, particle size, globule size, and rate/extent of drug release/permeation, are evaluated to ensure the desired product quality. However, alterations in these CQAs of the drug products may occur under ‘in use’ conditions because of various metamorphosis events, such as evaporation that leads to supersaturation and crystallization, which may eventually result in specific failure modes of semisolid products. Under ‘in use’ conditions, a limited amount of formulation is applied to the skin, where physicochemical characteristics of the formulation are substantially altered from primary state to secondary and, eventually, tertiary state on the skin. There is an urgent need to understand the behavior of topical semisolid products under ‘in use’ conditions. In this review, we attempt to cover a series of metamorphosis events and their impact on CQAs (Q3 attributes), such as viscosity, drug activity, particle size, globule size, and drug release/permeation of topical semisolid products.

## 1. Introduction

As an alternative to oral and intravenous delivery, topical drug delivery systems can transport the active drug molecules through the skin into the therapeutic target and transdermally into the circulatory system [1,2]. Typically, the most common topical products are in semisolid dosage forms, which include creams, ointments, gels, lotions, and emulsions [3]. Topical semisolid products enable the administration of active pharmaceutical ingredients (APIs) in a less invasive approach than the intravenous route and avoid the first-pass effect encountered by oral products, where drug molecules degrade to their inactive metabolites [4,5]. To achieve a desired therapeutic effect, these topical formulations must enhance the permeation of various drug molecules through the skin and maintain the rate and extent of penetration appropriately in the skin layers. The administration of drugs via topical drug delivery depends on various properties, such as physicochemical characteristics of the drug, interaction between excipients in the formulation and the skin, interaction of the drug with skin, interaction of the excipients with the drug, and an overall interplay of these three aspects, i.e., skin, drug, and excipients [6,7]. Moreover, drug penetration also depends on the skin condition, either diseased or normal, because in a diseased condition, the pathological state varies the bioavailability of the drug in skin layers. Stratum corneum (SC), the outermost layer of the epidermis, is composed of 85% of keratin-filled corneocytes and is a hydrophobic skin barrier that prevents the passive diffusion of large-sized molecules (>500 Dalton) [8,9]. Currently, in most commercially available topical semisolid products, their target sites are either the skin or subcutaneous tissue, and only a limited amount of the drug can be estimated in the systemic circulation. Thus, traditional approaches to demonstrate the bioequivalence (BE) and bioavailability (BA) of topical drugs usually involve clinical endpoint studies with pharmacokinetics, which are expensive and time consuming. Further, the data show high variability and poor sensitivity [10]. These conditions have become a barrier that limits the development of affordable topical drugs and abbreviated new drug applications (ANDAs). Therefore, to provide patients with high-quality and affordable products, the regulatory authorities are constantly seeking in vitro BE methods for complex topical drugs to waive in vivo BE studies for specific drug products that are Q1 (Qualitative Sameness), Q2 (Quantitative Sameness), and Q3 (Microstructural Sameness) to the reference list drugs [11].

Recently, the United States Food and Drug Administration (US FDA) updated and released new product-specific guidance for several drugs, in which in vitro options are given to the pharmaceutical industry. In general, the in vitro option consists of Q1/Q2 assessment, physiochemical characterization of formulations (Q3), and in vitro drug release and permeation tests. In these tests, the drug products’ critical quality attributes (CQAs), including particle/globule size, rheological properties, polymorphism, and formulation stability, are evaluated to ensure the desired product quality and drug performance [6]. A detailed list of APIs that have in vitro options in product-specific guidance is shown in Table 1. However, alterations in these CQAs of drug products may occur under the ‘in use’ condition due to metamorphosis caused by product dispensing, application to the skin, and evaporation. The series of events during metamorphosis can change the therapeutic profile and eventually lead to failure modes for semisolid products.

In the case of complex preparations, such as topical semisolid products, the formulation’s metamorphosis (e.g., evaporation, supersaturation, and crystallization) significantly impacts the drug products’ bioavailability. Depending on the dosage form, a range of CQAs can be changed when the topical products enter the metamorphosis, leading to specific failure modes, i.e., changes in particle size, globule size, rheological properties, and thermodynamic activity. In this review, we will focus on identifying changes in CQAs of topical semisolid products under ‘in use’ conditions by investigating the related series of metamorphosis events that can alter the drug performance and overall product therapeutic efficacy. In addition, various existing methods and potential techniques can elaborate on the relationship between these changes in CQAs and drug performance to better understand the possible failure modes of topical semisolid products.

## 2. Evaporation

Volatile excipients, such as water, ethanol, and propylene glycol, are commonly used as drug vehicles in marketed topical products. According to the topical dosage form nomenclature, different dosage forms of topical products can be determined based on the relative composition of volatiles [12]. The evaporation rate of these dosage forms (e.g., solution, suspension, lotion, gel, cream, ointment, and shampoo) varies with the different concentrations of volatiles. For example, gel evaporation occurs more rapidly than an ointment or a cream due to a higher proportion of volatiles, such as water and alcohol. In gel formulations for cold therapy, evaporation of methanol is utilized to provide a cooling sensation to relieve the inflammatory response and pain caused by soft tissue damage [13].

One of the predominant metamorphosis events of topical semisolid drug products is the evaporation of the vehicle’s volatile excipient in the formulation after application onto the skin. Surber and Knie classified the topical formulation into three stages, depending on the metamorphosis of drug vehicles: (1) primary formulation, which refers to the original formulation depositing in the primary container; (2) secondary formulation, which refers to the applied formulation during the dynamic stage of losing volatile vehicle ingredients; and (3) tertiary formulation, which refers to the final formulation with the evaporation of all volatile ingredients from the applied product on top of the skin [14].

During the shifting in these three stages, the loss of volatiles can lead to supersaturation of the formulation system, crystallization of the drug particles, alteration in the thermodynamic activity of APIs, and viscosity of formulations. All these alterations result in changes in product profile and drug performance [15]. It is challenging to differentiate the evaporation of water and volatile cosolvents by gravimetrical analysis methods, such as the measurement of the weight loss over time with a microbalance or thermogravimetric analyzer. For this, the literature disclosed the latest developed method to measure the loss of water using a customized evaporimeter directly and to compare the water loss to the weight loss of other volatiles in cream, gel, and lotion [16]. The findings demonstrated that the weight loss of water was smaller than the total weight loss of cosolvents in gels and lotions, suggesting a different evaporation rate of water and other volatile cosolvents. Therefore, water loss and overall product performance are significant for an effective and successful topical product. The detailed discussion related to metamorphosis is further described in the supersaturation section. Further, water activity measurement has become one of the standard comparative tests to characterize generic drugs and reference-listed drugs’ (RLDs) in vitro options available in product-specific guidance.

## 3. Supersaturation

There are various topical products in which APIs are dissolvable in volatile and non-volatile vehicles. The evaporation of volatile drug vehicles, such as acetone, methanol, and water, drives the drug’s sub-saturated or completely saturated systems to the supersaturated phase. To exemplify, once the product is applied on the skin, the volatile solvents in the formulation start to evaporate and reduce the drug solubility in the residues, resulting in higher thermodynamic activity and increased penetration of drugs into the skin.

Several attempts have been made to investigate the relationship between topical formulations’ supersaturation and thermodynamic activity. Santosh et al. studied the diffusion of fentanyl in propylene glycol/water solution (40:60 *v*/*v*) and propylene glycol/ethanol solution (40:60 *v*/*v*) from 1 degree of saturation to 7 degrees of saturation, under both finite and infinite dose conditions using silicone membranes, which are inert to the cosolvents [17]. A correlation between the penetration enhancement and degree of saturation was observed in propylene glycol/water formulations and propylene glycol/ethanol formulations under both dose conditions. The permeation enhancement ratio in propylene glycol/water formulations was significantly higher at infinite doses than at finite doses. The findings revealed that the higher flux in propylene glycol/ethanol formulations resulted due to an improvement in thermodynamic activity, triggered by the evaporation of ethanol. In a later study, they investigated the influence of different penetration enhancers, including propylene glycol, octyl salicylate, and isopropyl myristate, on the permeation of fentanyl using ex vivo human skin [18]. The findings of this study exhibited higher permeability from octyl salicylate and isopropyl myristate formulations due to supersaturation. However, this trend was not observed in the case of propylene glycol formulations because the formation of fentanyl crystals restricted the permeability due to the reduction in propylene glycol during metamorphosis events. Moreover, these studies provided evidence that favors rate of evaporation as a determinant of either supersaturation or crystallization.

In another study, Pellett et al. used full-thickness human skin to study the penetration of piroxicam in propylene glycol/water solution (40:60 *v*/*v*) from 0.5 degrees of saturation to 4 degrees of saturation [19]. In vitro permeation studies revealed a proportional relationship between the flux of piroxicam and the degree of saturation. Additionally, it was shown that supersaturated solutions at 4 degrees remained stable for 16 h, which can be attributed to the delay in the nucleation of piroxicam crystals by the lipids in the stratum corneum. Further, it was also observed that the amount of drug in viable layers increased when the degree of saturation increased and this amount of drug in the layers was determined by the tape-stripping technique [20]. Further, another group, Poulsen et al., demonstrated that the release of fluocinolone acetonide depended on the solubility and drug concentration in gel formulations [21]. Their findings revealed that the release of the drug into the receptor was significantly influenced by the solubility and partition coefficient of a drug. Therefore, the extent of solubility of APIs in vehicle is influenced and readily varied by metamorphosis, thereby potentially influencing the overall flux in the skin layers. In another case, Coldman et al. observed 8- to 10-fold higher penetration of fluocinolone acetonide in formulations containing isopropanol compared to formulations containing non-volatile vehicles because of supersaturation of fluocinolone in the formulation, which resulted due to the evaporation of isopropanol [22]. Furthermore, Theeuwes et al. compared the permeation of saturated and supersaturated solutions of hydrocortisone with acetone and water as drug vehicles. The results exhibited that the flux was significantly reduced from the initiation of nucleation in a supersaturated hydrocortisone solution at 560th minute. This process affected the overall permeability of the drug by altering the flux [23].

## 4. Crystallization

As reported by many researchers, maximizing the % saturation of APIs (i.e., thermodynamic activity) is as crucial as maintaining APIs dissolved in vehicles to optimize skin delivery for topical formulations [6]. Topical products, such as gels containing volatile vehicles of water and ethanol, can dry up shortly after application onto the skin, resulting in a thermodynamically unstable supersaturated system. In this case, APIs will spontaneously crystallize, followed by the inevitable nucleation. In a study, Barrett et al. studied the percutaneous absorption of the crystalline form of fluocinolone acetonide and its solution. They found that fluocinolone acetonide solution dissolved in the soft white paraffin had a higher degree of vasoconstriction than its crystalline form [24]. Chia-Ming et al. observed a reduction in the flux of minoxidil at various concentrations (3%, 4%, and 5%) in formulations composed of propylene glycol, ethanol, and water. The decrease in flux was attributed to the crystallization after the evaporation of vehicles [25]. Apart from the evaporation of cosolvents, temperature fluctuation, pH change, and impurities can also lead to the growth of crystals. Further, the findings showed that the permeability was drastically reduced due to the formation of drug crystals. In addition, the inconsistent plasma drug concentration was observed due to the change in the morphology of crystals over time in the course of metamorphosis events. Therefore, the change in the morphology sometimes leads to the formation of the desired drug morphology required for the permeation across the skin barrier. Considering the above findings, the inconsistent plasma drug concentration is considered a potential failure mode of topical products.

To tackle these failure modes caused by the instability and drug crystallization, antinucleating agents, such as hydroxypropyl methylcellulose, polyvinyl alcohol, and polyethylene glycol, are often used. These polymers can maintain the formulation at a high level of drug activity and mitigate the failure mode by delaying the formation of the nucleus or modifying the morphology and retarding the growth of crystals. To illustrate, Iervolino et al. reported a slower growth rate of ibuprofen crystals in five-times supersaturation propylene glycol/water solution with 2-hydroxypropyl-β-cyclodextrin or hydroxypropyl methylcellulose than in solution without any additives [26].

**Figure 1:**
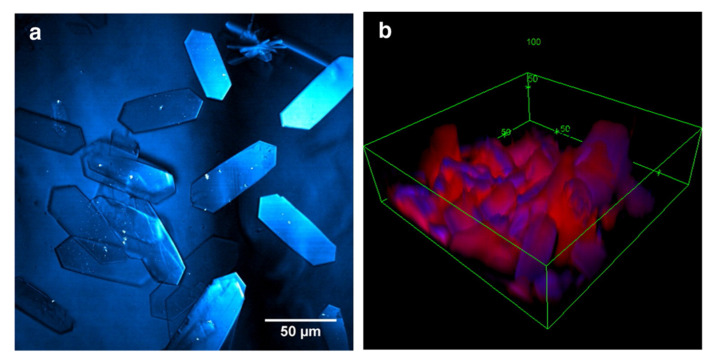
Crystals of Ibuprofen-d3 formed after 30 min of application onto the skin; (**a**) topology of the crystals on the skin; (**b**) 3D representation in which ibuprofen represented in blue and skin lipids false-colored in red (figures reproduced with permission from Belsey and associates [28].

Furthermore, Raman spectroscopy has been widely used to distinguish the chemical distribution of topical formulations applied on the skin with label-free chemical maps by analyzing the spectra acquired per pixel. However, the acquisition time of individual frames can be expensive and time consuming for helpful resolution. Therefore, Stimulative Raman Scattering (SRS), an emerging non-linear microscopy that uses both pump beam and Stokes beam to excite the objects, reduces the imaging time and backscattering in spontaneous confocal Raman spectroscopy, thus, providing an opportunity to quantify the penetration of APIs into skin layers and elucidate the metamorphosis of topical formulations [27]. As shown in Figure 1, Belsey et al. captured the image of Ibuprofen-d3 crystals on the skin after applying its sub-saturated solution in propylene glycol for 30 min using SRS [28]. Additionally, a similar crystallization process of ketoprofen was observed in their studies, revealing the metamorphosis of formulations. Saar et al. also successfully visualized the growth of ibuprofen crystals on mice ear tissue’s surface and hair shaft [29]. By tuning the intensity and frequency of both the pump and Stokes beam, the state-of-the-art SRS allows video-rate imaging to study the in situ percutaneous absorptions of APIs and vehicles and crystallization [30].

## 5. Viscosity

Viscosity is one of the most critical parameters and is considered a CQA for the development of various topical products. The inability to achieve the appropriate and desired viscosity index during the development of the formulation can lead to failure modes of the drug under ‘in use’ conditions. For instance, the solvent mixture is trapped in the three-dimensional network of linked polymer chains in hydrogels and the polymeric content rises due to the evaporation of the volatile solvent. Additionally, a denser gel network may occur in carbomer gels with two different polymeric levels because of simultaneous evaporation and absorption of a vehicle into the skin. Therefore, the rearrangement and deformation of product structure can alter the rheological properties of topical products applied to the skin.

Cross et al. studied the penetration of oxybenzone emulsions containing the thickening agent carbomer 940 (from 0% to 0.5%), under both infinite-dose (static) and finite-dose (in-use) conditions across human skin [31]. The water loss of four emulsion formulations was estimated to understand the evaporation rate. Additionally, a Brookfield viscometer determined the viscosity of four formulations with a shear rate of 0.3 RPM. The skin retention of oxybenzone was compared to illustrate the difference in the drug penetration process of viscous formulations under two dosing conditions. It was observed that the flux was inversely proportional to the viscosity of formulation under the infinite-dose condition, while the flux increased along with the increment in viscosity for emulsions under the finite-dose condition. The authors also highlighted the viscosities’ change before and after membrane diffusion studies due to a higher evaporation rate for the ‘in-use’ situation.

**Figure 2:**
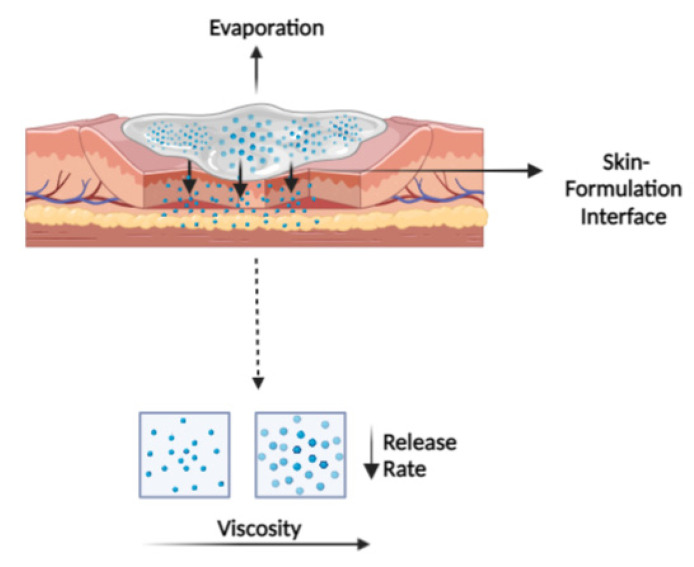
The graphical illustration of the interplay between the applied formulation and skin. The viscosity of topical semisolid products increased with the evaporation of volatile solvents, resulting in a more compact product microstructure at the skin–formulation interface. Thus, the release rate of APIs from the formulation was significantly reduced, indicating the product’s lower permeability and overall therapeutic efficacy.

As illustrated in Figure 2, the metamorphosis drastically impacts the viscosity and polymeric or hydrocolloid network of topical semisolid products applied on the skin and, thereby, the drug flux. Therefore, it is essential to understand the relationship between drug release/penetration profile and viscosity. To understand this, Binder et al. established a correlation between the viscosity and permeability of sulphadiazine sodium hydrogels with two different gelling agents, i.e., hydroxyethyl cellulose or hydroxypropyl methylcellulose [32]. The results of this study demonstrated a significant decrease in release rate, penetration rate, and depth, with an increase in viscosity, suggesting a retardant effect on drug penetration. This is due to a dense gel network formed with the evaporation of drug vehicles. Apart from this, spreading and skin coverage of formulations with different viscosities contribute to additional failure modes.

## 6. Thermodynamic Activity

During the metamorphosis process, primary formulation changes to secondary and tertiary formulation after application onto the skin. This results in an increase in API concentration in the formulation and the evaporation of volatile drug vehicles. As noted in previous sections, it reaches the level of saturation and possibly supersaturation on the skin surface; when the thermodynamic activity of APIs in topical formulations changes, it exhibits different skin-penetration profiles.

Under ideal conditions, the passive diffusion process of drug permeation can be described by Fick’s first law:

$$Jss=-D\frac{dc}{dx}$$

where *Jss* is steady state flux, *D* is diffusion coefficient, *C* is concentration, and *x* is distance. It is apparent that steady-state flux is proportional to the drug concentration for an ideal mixture. However, most topical drug products behave non-ideally. Thus, the passive diffusion process is governed by the gradient of chemical potential (instead of concentration) of drug substances, which is determined by its thermodynamic activity (i.e., effective concentration). The relationship between the thermodynamic activity (a) of a drug substance and its chemical potential (*μ*) can be described with its standard potential (*μ*°), gas constant (*R*), and temperature (*T*) [33]:

μ=μ°+RTlna

An equation for thermodynamic activity firstly expressed the steady-state flux by Higuchi in 1960:

$$Jss=\frac{Da}{\gamma h}$$

where *γ* is activity coefficient and *h* is thickness of membrane [34]. The relative activity of a drug substance can be presented as its % saturation (i.e., the ratio of drug concentration in the formulation to the concentration at saturated status). Upon the evaporation of volatile cosolvents, the % saturation of APIs increases on the skin after applying topical semisolid products, resulting in a change in the thermodynamic activity of APIs in the mixture system and, thereby, the permeability.

Barry et al. investigated the diffusion process of benzyl alcohol through human skin to correlate the flux and thermodynamic activity [35]. In their study, the vapor pressure and concentration of benzyl alcohol in binary mixtures constituted with selected vehicles, including toluene, butanol, butyl acetate, isopropyl myristate, isophorone, and propylene carbonate, were measured by headspace analysis to determine the thermodynamic activity of benzyl alcohol. The non-ideal behavior of benzyl alcohol was identified in these binary mixtures. The benzyl alcohol vapor flux through human skin was measured with vapor diffusion experiments, and a linear relationship between the thermodynamic activity of benzyl alcohol and its vapor flux was identified. Their later study also reported this linear relationship of liquid benzyl alcohol [36]. However, these studies were limited to binary mixtures and multiphase formulations (creams and ointments) were not considered.

Kokate et al. studied the permeation profile of two ionizable drugs, nimesulide and bupivacaine, under different pH to investigate the thermodynamic activity of ionized and unionized drug species in the permeation process [37]. The saturated solubility of two drugs in buffer solutions of a series of pH values was studied with the shaking-flask method. The friction of ionized and unionized species was calculated with Henderson–Hasselbalch equation to determine the degree of saturation of ionized and unionized species. The permeation profile of nimesulide and bupivacaine under different pH and concentrations was studied using porcine buccal mucosa. It can be concluded from this study that the thermodynamic activity of both ionized and unionized species contributes to the permeation of ionizable drugs, indicating the potential influence of pH change and solubility of drug molecules’ change in the topical formulation.

Roberts et al. studied the permeability of phenolic compounds across the human epidermis. It was observed that the permeability was related to the partition coefficient of phenolic compounds, and the skin penetration rate was dependent on the solubility and concentration of lipophilic molecules [38]. Furthermore, Zhang et al. also reported that the maximum flux of phenols and their solubilities in the stratum corneum is proportional to the octanol–water partition coefficient [39]. Similar trends for ten different similar-sized phenolic compounds were also observed in their later study, revealing the determinant of thermodynamic activity [40]. Anissimov and Roberts established a diffusion model to demonstrate the percutaneous absorption of solute under finite-dosing conditions, where the solute’s saturation and solubility were relevant to the steady state of flux [41].

## 7. Microstructural Change

The CQAs, such as particle size and globule size, are important for topical drug products, especially for those formulations with APIs suspended in semisolid dosage forms. For topical drug products containing volatile vehicles, the concentration of API increases in the formulation along with the evaporation of volatile vehicles and, eventually, drug crystals may grow [42]. It is also apparent that the efficacy of skin penetration depends on the size of particles because it affects the penetration pathways (i.e., transcellular, intercellular, and transappendageal routes) of smaller particles [43,44]. For transappendageal routes, small drug particles pass the stratum corneum through hair follicles and, for transcellular routes, the dissolved drug directly penetrates through the corneocytes, while intercellular penetration involves the movement of drug molecules along the lipids located at intercellular spaces of corneocytes. Moreover, particle size is directly related to dissolution of drug particles. Ideally, it is desirable to formulate with the dissolution rate of particulate drugs, higher than the transport flux through the stratum corneum. This ensures a steady near-saturated concentration in the formulation. Thus, the particle size impacts the percutaneous absorption, as small particles (nanoparticles) need to fit into the space between corneocytes and migrate along the gap. To understand the penetration pathway, Sirirak reported the transappendageal pathway as the predominant route for the skin penetration of celecoxib-loaded microemulsion, with particle sizes ranging from 48 to 214 nm [45]. The penetration can be facilitated for particles with higher partition coefficients (log P) due to their higher solubility in the stratum corneum, which is a lipophilic skin barrier. In another case, Lademann et al. also reported the difference in reservoir behavior of the stratum corneum and hair follicles, as a longer storage time of drugs was observed in hair follicles than in the stratum corneum [46]. Therefore, the change in particle size distribution in topical products, which can be caused by aggregation of particles, phase separation of emulsions, and polymorphism, can potentially alter bioavailability. Additionally, the rate of dissolution, which is a determining factor in semisolids with suspended API, is significantly altered for different-sized particles. These complex formulations require the rate of dissolution of crystals to be the same or higher than the rate of penetration to maintain a constant thermodynamic potential.

Globule size is an important factor for dosage forms, such as emulsions, not only due to its effects on shelf life and stability but also because it determines the release rate and extent of APIs, i.e., flux and cumulative amount from the formulation into the skin. It depends on the droplet volume ratio to the emulsion volume, i.e., the dispersed-phase volume fraction [47]. In addition, changes in globule size can lead to a shift in drug partitioning between the dispersed phase and continuous phase for formulations with drug molecules dissolvable in both phases. The surface area contacted with skin increases with the decrease in globule size in emulsions, resulting in higher drug adsorption. Patel et al. found a higher drug permeation in microemulsions with smaller globule sizes, leading to a higher drug concentration in the subcutaneous tissue and more efficient drug delivery [48].

Thus, considering all these parameters, dermal drug delivery can be significantly influenced by particle and globule size distribution after the product application onto the skin. Therefore, it is necessary to have robust methods for the measurement of particle and globule size. The selection of a method to determine the size of the particle and globule depends on the dosage form and morphology; therefore, methods should be chosen carefully with respect to the dosage form and structure. Various methods are tabulated in Table 2 to determine the size in the nanometer range to the micrometer. We also mentioned the estimation range of methods and their advantages and disadvantages.

Jung et al. used confocal Raman microscopy to study the impact of mechanical stress applied to the acyclovir cream (Zovirax^®^ UK pump) during product dispensing on the physiochemical property of products [49]. The findings revealed blue round droplets, identified by Raman spectroscopy as dimethicone, were found in the cream base that underwent deformation during pumping. They hypothesized that the rearrangement of ingredients in the topical product and the microstructural change, i.e., dimethicone separation, can form a barrier at the formulation–skin interface and adversely affect the skin penetration of API. In the end, a lower cumulative amount of acyclovir was reported in the formulation dispensed by the pump, as compared to the topical application of acyclovir cream.

## 8. Skin Permeation Studies

CQAs of topical semisolid products can be altered because of metamorphosis under ‘in use’ conditions. Thus, it is essential to establish methods that can decipher the impact of these changes on skin penetration. There is no commercially available equipment entirely suited to study the topical formulation under the “in use” experiment setting. To develop these semisolid topical products, it is important to understand their penetration profiles and evaluate the bioavailability of different formulations by assessing the local skin pharmacokinetics. The clinical endpoint study approach studies products under in-use conditions; however, it is expensive and time consuming. Therefore, various alternative methods, including microdialysis [50], tape-stripping experiment (dermatopharmacokinetics) [51], in vitro permeation testing (IVPT) [52], and confocal Raman microscopy [53], are being developed and optimized to study topical drug penetration.

Among these techniques, IVPT is a standardized test accepted by the Organization for Economic Cooperation and Development (OECD) in test guideline 428 and Product-Specific Guidance for Generic Drug Development. This test is carried out to predict the in vivo absorption by studying the rate and extent of skin penetration of topical formulations with suitable ex vivo animal or human skin membranes. Nowadays, artificial membranes, such as Strat-M™ and reconstructed human skin models (cell culture based), are also applied to in vitro releasing testing (IVRT) studies because of ethical considerations and regulatory requirements in different countries and areas. To depict various skin models, a range of skin models is discussed in Table 3.

**Figure 3:**
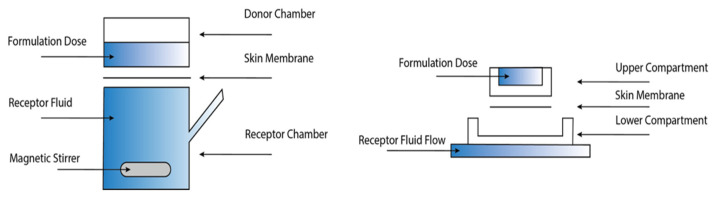
Schematic diagram of vertical diffusion cells (left: Franz cells; right: flow-through cells).

Vertical diffusion cells are used in IVPT/IVRT studies to analyze the permeation of active drug compounds through semipermeable membranes by quantifying the drug flux over time, related to the concentration of permeants in the receptor medium. Several commercially available versions are allowed by regulatory agencies. However, most versions have an enclosed donor chamber, resulting in airflow and evaporation dynamics in the chambers being entirely different. This has the potential to cause inter-lab variability to the absolute penetration data. Combined with personnel skill and expertise, the overall difference in data generated in different labs can be sizable and, at times, significant. As illustrated in the schematic below (diffusion cell assembly in Figure 3), the skin membrane is sandwiched between the donor chamber (upper compartment) and receptor chamber (lower) in most designs of vertical diffusion cells. The testing formulation is dosed on top of the skin in the donor chamber. At the same time, the receptor medium is filled in the receptor chamber and constantly withdrawn manually or automatically. It is advisable that, to maintain the sink condition (i.e., dissolving drug molecules in the receptor medium) and reduce the possibility of back diffusion throughout the membrane, the saturated solubility of permeants in the receptor medium should be ten-times higher than the concentration of permeants in the receptor medium during experiments [56].

Tom Franz, in 1975, validated the use of static glass diffusion cells to predict the in vivo percutaneous absorption and developed Franz cells [57]. In static Franz cells, magnetic stir mini bars are placed in receptor chambers to stir the receptor medium, which is collected and replaced with fresh medium at sampling time points. The experiment settings in IVPT studies using Franz cells include receptor volume, drug clearance, dosing area, chimney height of the donor chamber, etc. With the development of additive manufacturing technology, Sil et al. fabricated resin-based Franz cells with a 3D printer, allowing rapid and cost-effective customization of the IVPT experiment apparatus to meet optimized experiment settings [58,59]. However, no current model assesses penetration in a clinical-like setup. Over close to 50 years, there have only been a handful of attempts to develop diffusion cell donor chambers that mimic clinical settings. Wagner et al. established an in vivo/in vitro correlation for skin penetration of flufenamic acid dissolved in wool alcohol ointment by determination of the drug content in the stratum corneum and deeper skin layers of the same skin sample using Finn chambers for in vivo study and Saarbruecken penetration model/Franz cells for in vitro study [60]. A linear correlation of drug content between in vivo and in vitro was established for the deeper skin layers. A similar relationship was also observed in their earlier studies for the stratum corneum [61]. The other design of vertical diffusion cells is flow-through cells. In flow-through cells, the medium in the receptor chamber is continuously refreshed with a constant flow from the peristaltic or syringe pump connected to the reservoir of the receptor medium. It is desired that sampling at the initial stages of IVPT experiments is as frequent as possible to accurately quantify initial pharmacokinetics (i.e., permeability coefficient (Kp) and lag time (t_lag_)) [62]. Therefore, a higher flow rate may be required to produce a sufficient volume of sample and maintain the sync condition. This, in turn, as a result of excessive dilution, can potentially induce the detection limit problem of permeants with slow release/penetration rate [63]. Thus, the clearance of flow-through cells, i.e., a ratio of flow rate and receptor volume, is one of the critical parameters, which often needs to be optimized to prevent detection limit problems in the earlier phase of the IVPT experiment circle. Tanojo et al. designed a novel flow-through cell with a spiral receptor chamber to reduce the influence of receptor volume and stagnant domains in the receptor chamber [64].

In the experimental set-up, there are two scenarios under which IVPT studies are performed, infinite dosage and finite dosage. For infinite dosing, a large amount of testing formulation is applied to the skin. The formulation loss caused by evaporation and skin absorption is considered negligible compared to the dosing amount. For semisolid products, a crust formation in the top layer evaporated also delays further metamorphosis. Under finite dosing, only a limited amount of formulation (smaller than 10 μL/cm²) is applied to the skin to mimic the in-use conditions [56]. To predict the “in use” penetration profile of metronidazole semisolid products under clinical conditions, Arora et al. developed a physiologically based pharmacokinetic model of metronidazole using IVPT data, which successfully captured the metamorphosis of metronidazole gel and cream after application [65]. The influence of evaporation of volatiles and gradual change in vehicle composition on skin penetration can be, thereby, observed. Chen et al. reported the difference in skin penetration and drug deposition for hydrophilic drugs between two dosing scenarios due to the effect on the hydration status of the stratum corneum [66]. The pH change under finite-dose conditions should also be taken into consideration, as the pH partition theory suggests the effect of ionization status (ionized, unionized, ion paired) of ionizable drugs on skin penetration [67,68,69]. As predominant drugs that permeate the skin are in un-ionized form, the permeability of the ionizable drugs, such as weak acids or bases, can be changed due to pH change of formulations applied onto the skin as a result of metamorphosis and buffering capacity of skin.

## 9. Conclusions

In this review, we covered a series of metamorphosis events, such as evaporation, and their impacts on CQAs (viscosity, thermodynamic activity, particle size, globule size, and drug release/permeation profile) of topical semisolid products. These metamorphosis events are interconnected with each other. For example, water evaporation and volatiles in topical formulations can lead to supersaturation and crystallization. As the manifestation of metamorphosis, the changes in CQAs will alter drug performance or even become potential failure modes of topical products. The growing list of product-specific guidance that has in vitro BE options for developing generic topical semisolid drugs requires the respective technologies to study these CQAs. However, our understanding of the dynamic process of percutaneous absorption and metamorphosis is far behind ‘in use’ condition. Thus, there is an urgent need to develop robust methods to correlate changes in CQAs with metamorphosis events.

## Figures and Tables

**Table 1:** The data were obtained from US FDA databases. List of APIs approved for topical drug products in semisolid dosage forms acquired from Orange Book: Approved Drug Products with Therapeutic Equivalence Evaluations before APIs filtered by the Product Specific Guidelines database for interrogation of in vitro characterization options.

APIs	Dosage Form	Qualitative and Quantitative Assessment (Q1 & Q2)	Comparative Physicochemical Characterization (Q3)	In Vitro Release Testing (IVRT)	In Vitro Permeation Testing (IVPT)	Additional In Vivo Pharmacokinetic Study	Year
Acyclovir	Ointment	+	+	+			2019
Acyclovir	Cream	+	+	+	+		2016
Bexarotene	Gel	+	+	+			2019
Clindamycin phosphate	Gel	+	+	+			2020
Crisaborole	Ointment	+	+	+	+	+	2019
Dapsone	Gel	+	+	+	+	+	2019
Docosanol	Cream	+	+	+			2017
Doxepin hydrochloride	Cream	+	+	+	+	+	2019
Erythromycin	Gel		+				2019
Fluocinolone acetonide	Cream		+				2018
Gentamicin sulfate	Ointment		+				2017
Gentamicin sulfate	Cream		+				2017
Hydrocortisone	Cream		+				2017
Ivermectin	Cream	+	+	+	+	+	2019
Lidocaine	Ointment	+	+				2016
Luliconazole	Cream	+	+	+	+		2018
Metronidazole	Gel	+	+	+			2019
Metronidazole	Cream	+	+	+	+		2019
Oxymetazoline hydrochloride	Cream	+	+	+	+		2019
Penciclovir	Cream	+	+	+	+		2018
Pimecrolimus	Cream	+	+	+	+		2019
Silver sulfadiazine	Cream	+	+	+			2017
Tacrolimus	Ointment	+	+	+	+		2018
Tretinoin	Gel	+	+	+			2020
Triamcinolone acetonide	Cream		+				2017

**Table 2:** Available techniques to determine the particle size.

NO.	Available Techniques	Working Range	Advantages	Disadvantage
1	Dynamic Light Scattering	10 nm–10 µm	Smaller sample volume	Need of sample dilution, unsuitable for viscous samples.
2	Laser Diffraction	10 nm–1 mm	Reproducible, smaller sample volume, suitable for spherical particles.	Inaccurate results for irregularly shaped particles need sample dilution.
3	Morphologically Directed Raman Spectroscopy (MDRS)	1 μm–1 mm	Spectroscopic interrogation of particles.	Shorter working range.
4	Optical microscopy (bright-field microscopy and polarized light microscopy)	1 μm–1 mm	Rapid identification of drug crystals.	Shorter working range, sensitive to sample preparation.

**Table 3:** Skin models used in IVPT and IVRT studies.

**NO.**	**Skin Models**	**Examples**	**Advantages**	**Disadvantages**
1	Human skin models	Full-thickness human skin, dermatomed human skin, the epidermis.	Anatomically identical to human in vivo skin	Ethical considerations, Inconsistency between skin donors
2	Animal skin models	Rats skin, snakeskin, porcine skin, macaque skin.	Histologically similar to human skin	More permeable than ex vivo skin
3	Reconstructed human skin equivalents	Reconstructed full-thickness skin, the reconstructed epidermis (EpiSkin^®^, SkinEthic^®^, EpiDerm^®^).	Structurally close to human skin	More permeable than ex vivo skin
4	Synthetic membranes	Strat-M™, parallel artificial membrane permeability assay [54], phospholipid vesicle-based permeation assay [55].	Reproducible and consistent results	Differences in lipid compositions to the human skin
